# The effect of radiotherapy time and dose on acute hematologic toxicity during concurrent postoperative chemoradiotherapy for early high-risk cervical cancer

**DOI:** 10.7150/jca.82801

**Published:** 2023-04-01

**Authors:** Xiaojing Yang, Zhen Li, Lihua Zhang, Hongling Li, Xinmiao Yang, Yi Sun, Lijun Liu, Jie Fu

**Affiliations:** Department of Radiation Oncology, Shanghai Sixth People's Hospital Affiliated to Shanghai Jiao Tong University School of Medicine, Shanghai, China

**Keywords:** Early-stage high-risk cervical cancer, Concurrent chemoradiotherapy, Acute hematological toxicity, Pelvic bone marrow dose volume

## Abstract

**Objective:** This study aims to analyze the characteristics and factors that influence acute hematological toxicity (HT) during concurrent chemoradiotherapy (CCRT) for cervical cancer, as well as to provide reference data for clinical practice.

**Methods:** Patients with FIGO IB1-IIA2 cervical cancer who underwent CCRT from May 2018 to August 2020 were included in this study retrospectively. All patients had received external beam radiation therapy and platinum-based concurrent chemotherapy. HT was assessed according to CTCAE 5.0. The pelvic bone marrow was redrawn on the original CT images and divided into four parts: the whole pelvic bone marrow (WP-BM), iliac bone marrow (IL-BM), lower pelvic bone marrow (LP-BM), and lumbosacral bone marrow (LS-BM). The radiation dose and volume of each part of the pelvic bone marrow were recalculated in a new plan created using the original planning parameters. The corresponding dose-volume histogram (DVH) was generated to obtain the bone marrow volumes receiving 10Gy, 20Gy, 30Gy, 40Gy, 45Gy, and 50Gy.

**Results:** In 112 patients, the incidences of grade 2 or higher leukopenia, anemia, thrombocytopenia, and neutropenia were 49.1%, 2.7%, 1.8%, and 20.5%, respectively. Leukopenia was linked to LS-V20 (*r* = -0.310; *P* = 0.006) and radiotherapy treatment lengths (days) (*r* = -0.416; *P* = 0.013). Anemia was associated with WP-V30, WP-V40, WP-V45, WP-V50, IL-V20, IL-V40, ILV45, IL-V50, LP-V30, LP-V40, LP-V45, and LP-V50 (*P* <0.05). Thrombocytopenia (r = -0.304, *P* = 0.007) and neutropenia (r = -0.368, *P* = 0.009) was associated only with the length of radiotherapy treatment (day). Multiple regression analysis showed that only anemia was negatively correlated with WP-V30, IL-V40, and LP-V40 (*P* <0.05).

**Conclusions:** Acute HT during CCRT in early-stage high-risk cervical cancer may be related to the duration of radiotherapy and the volume of different radiotherapy doses received at different parts in the pelvic bone marrow.

## Introduction

Cervical cancer is the most frequent gynecological malignancy, with the highest incidence in all gynecological cancers 1. The combination of radical hysterectomy and pelvic lymph node dissection is a radical treatment for early cervical cancer that has been proven to be effective 2. Studies have shown that for patients with cervical cancer without postoperative risk factors, the 20-year overall survival rates of radical radiotherapy and radical surgery are comparable, both exceeding 70% 3. However, studies have found that in patients with postoperative intermediate risk factors (tumor diameter > 4 cm, deep myometrial invasion of the cervix, or lymphovascular space invasion), the recurrence rate is lower if only one single intermediate risk factor exists. Moreover, the recurrence rate can reach 15%-20% if multiple intermediate risk factors coexist 4. However, the recurrence rate of patients can be as high as 25%-30% if postoperative high-risk factors exist (pelvic or para-aortic lymph node metastasis, positive surgical margins, or parametrial infiltration) 5. Therefore, postoperative adjuvant therapy is required to reduce recurrence and improve patient survival when risk factors are discovered after surgery in patients with early-stage cervical cancer. Studies have confirmed that for patients with intermediate or high risk, postoperative chemoradiotherapy can improve their prognosis 6, 7. Therefore, the role of concurrent chemoradiotherapy (CCRT) in postoperative adjuvant therapy has been established for early-stage cervical cancer with intermediate or high-risk factors.

CCRT has a significant curative effect in treating patients with early-stage high-risk cervical cancer. However, it still causes some acute adverse reactions, which mainly include acute hematology toxicity (HT), gastrointestinal toxicity, and genitourinary system toxicity, and acute HT has the highest incidence rate 8. Acute HT can lead to the interruption of radiotherapy and prolong the treatment time. It also reduces patients' chemotherapy tolerance and the number of chemotherapy cycles, resulting in an increase in patients' economic burden and poor treatment outcome 9, 10. As a result, a thorough investigation of HT's clinical features and influencing factors during CCRT is critical 11.

The results of studies on acute HT during CCRT for cervical cancer are currently inconsistent, as are the concurrent chemotherapy regimens. In addition, radiotherapy techniques are evolving at a rapid pace, necessitating more research in this area. This study retrospectively analyzed the influence factors of acute HT in postoperative radiotherapy combined with platinum-based concurrent chemotherapy in patients with early-stage high-risk cervical cancer. It provided theoretical guidance for the clinical treatment of early-stage high-risk cervical cancer patients after surgery.

## Materials and methods

### Patients

From May 2018 to August 2020, 112 patients with stage IB1-IIA2 cervical cancer who underwent radical concurrent chemoradiotherapy in our department were studied retrospectively. Patient data were collected from the medical history system and the Pinnacle3 treatment planning system (Version9.2, Philips Radiation Oncology Systems, Philips Healthcare). The following are the case inclusion criteria: ① Pathology confirmed cervical squamous cell carcinoma, adenocarcinoma, adenosquamous cell carcinoma, and small cell carcinoma; ② The International Federation of Gynecology and Obstetrics (FIGO) stage IB1-IIA2; ③ Existence of one or more high-risk factors (pelvic lymph node metastasis, positive resection margin, or parametrial invasion) or the coexistence of two or more intermediate-risk factors (tumor diameter > 4cm, intravascular tumor thrombus, or deep tumor Interstitial infiltration) revealed by the post-operative pathological examination; ④ ECOG (Eastern Cooperative Oncology Group) score of 0-2; ⑤ Sufficient bone marrow hematopoietic function (neutrophil count ≥ 1.5 × 10^9^/L, platelet count ≥ 100 × 10^9^/L); ⑥ No previous pelvic radiation therapy. All patients signed informed consent. The study was approved by the Ethics Committee of Shanghai Sixth People's Hospital Affiliated to Shanghai Jiao Tong University School of Medicine and followed the Declaration of Helsinki to the letter.

### Treatment

#### CT simulation

The patient was positioned in a supine position and fixed with a thermoplastic pelvic peritoneum. The CT scan was performed using a Siemens simulation CT with a bore size of 82 cm. CT scans patients from the upper border of the first lumbar vertebra to 5 cm below the sciatic tubercle. The slice thickness was 5 mm. Images acquired after scanning were transferred to Pinnacle3 treatment planning system (Version 9.2, Philips Radiation Oncology Systems, Philips Healthcare) for the delineation of the target and Organs at risk (OARs).

#### External beam radiation therapy

##### Targets and OARs delineation (Figure 1)

Target delineation was performed on CT images with reference to pelvic MRI. The Clinical Target Volume (CTV) ranges from the 4th-5th lumbar vertebrae (upper boundary) to the lower border of the obturator (lower boundary), including the tumor bed and pelvic lymphatic drainage area. The lymphatic drainage area includes parailiac vessels, presacral, and obturator pore lymphatic drainage area. The CTV might not cover the common iliac lymphatic drainage area if the pelvic lymph nodes were negative. If the common iliac lymph nodes are positive, the abdominal lymphatic drainage area should be included. The Planning target volume (PTV) was 5mm of CTV expansion. OARs include the spinal cord, bladder, femoral head (left and right), small intestine, rectum, and anus.

##### Treatment planning

The IMRT (Intensity-modulated Radiation Therapy) plan was completed using inverse planning in the Pinnacle3 treatment planning system. The prescription dose was set to be 1.8Gy per fraction and totally 45-50.4Gy in 25-28 fractions. The prescription dose should cover more than 95% of the PTV, and the maximum dose should be less than 110% of the prescription dose. Each patient performed 7 fields at the same center. Some patients underwent a boost with 6MV beam for local reduction of the vaginal stump, 6-MV-X-ray, IMRT, 10Gy/5Fx. The corresponding dose-volume histogram (DVH) was generated after planning for subsequent analysis.

##### Simulation and positioning

According to the calculation results of the IMRT planning system, the reference point of the setup was determined under the simulator (Nucletron Simulix), and the correctness of each irradiation field was verified.

##### Treatment delivery

Radiation treatment was delivered once a day, five times per week, using Siemens ARTISTE Linear Accelerator.

#### Concurrent platinum-based chemotherapy

Patients received a weekly intravenous cisplatin infusion of 40 mg/m^2^ or a weekly intravenous nedaplatin infusion of 30 mg/m^2^. All patients should take a blood routine examination before each cycle of chemotherapy. The chemotherapy will be postponed if the neutrophil count is less than 1.5 × 10^9^/L or the platelet count is less than 100 × 10^9^/L.

### Assessment of HT during treatment

The inhibition degree of four indicators (leukocytes, neutrophils, hemoglobin, and platelets) during treatment was assessed according to the CTCAE (Common Adverse Events Evaluation Criteria, version 5.0).

### Pelvic bone marrow delineation and dose calculation

**Figure 2:**
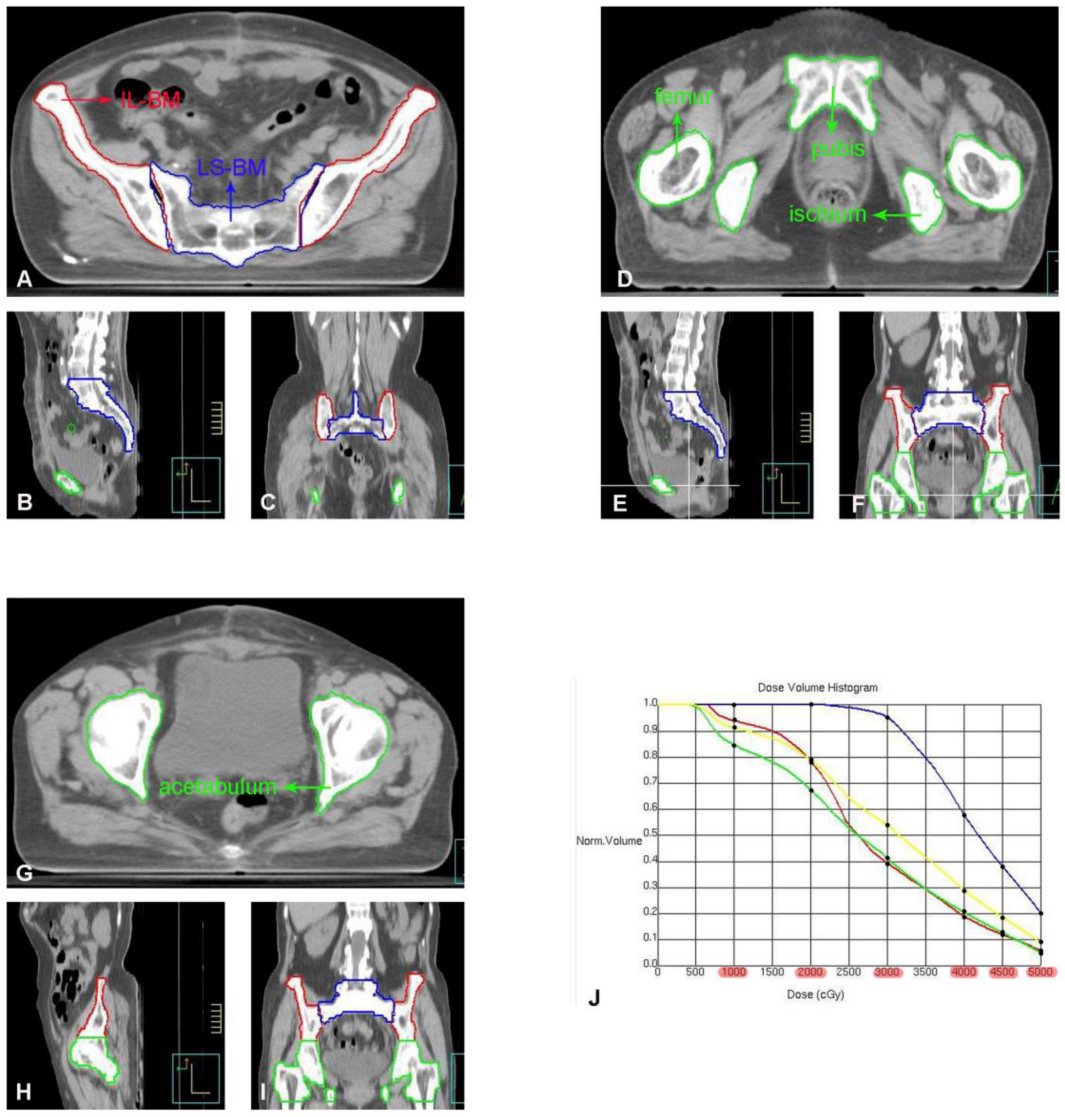
A-I: Each part of the pelvis bone marrow on the pelvic transverse section (A, D, G), sagittal plane (B, E, H) and coronal plane (C, F, I). J: DVH of each part of the pelvis bone marrow (IL-BM, LP-BM, LS-BM, and WP-BM) receiving the dose of 10Gy, 20Gy, 30Gy, 40Gy, 45Gy and 50Gy, respectively. The red line represents IL-BM. The green line represents the LP-BM including pubis, ischium, acetabulum and proximal femur. The blue line represents LS-BM. The yellow line represents WP-BM.

The pelvic bone marrow was redrawn on the original CT image of the patient and divided into four parts: ① Ilium Bone Marrow (IL-BM): from the level of the iliac ridge to the level of the upper edge of the femoral head; ② Lower pelvic bone marrow (IL-BM): from the level of the upper border of the femoral head to the level of the lower border of the ischial tuberosity, including the pubic bone marrow, ischial bone marrow, acetabular bone marrow, and proximal femoral bone marrow; ③ Lumbosacral Bone Marrow (LS-BM): including the fifth lumbar vertebra and the entire sacrum; ④ Whole pelvic bone marrow (WP-BM): including ①-③ (Figure 2). The radiation dose and volume of each pelvic bone marrow part were recalculated using a new plan created based on the original radiotherapy plan parameters. The corresponding DVH was generated (as shown in Figure 2) to obtain V10Gy, V20Gy, V30Gy, V40Gy, V45Gy, and V50Gy for each part of the pelvic bone marrow 12. These dose parameters were recorded as follows: ① Ilium bone marrow V10 (IL-V10), IL-V20, IL-V30, IL-V40, IL-V45, IL-V50; ② Lower pelvic bone marrow V10 (LP-V10), LP -V20, LP-V30, LP-V40, LP-V45, LP-V50; ③ Lumbar sacral bone marrow V10 (LS-V10), LS-V20, LS-V30, LS-V40, LS-V45, LS-V50; ④ Whole pelvic bone marrow V10 (WP-V10), WP-V20, WP-V30, WP-V40, WP-V45, WP-V50.

### Statistical analysis

Statistical analysis was performed using SPSS 24.0. The age of the patients in the two groups was tested by paired t-test. The Wilcoxon rank-sum test was used for the FIGO stage, radiation dose, and hematologic toxicity. Pathological types were tested by Fisher's exact test. The depth of interstitial invasion, intravascular tumor thrombus, and pelvic lymph node metastasis were tested by the χ^2^ test. Continuity-adjusted χ^2^ test was used for the resection margin, parametrial invasion, total number of high-risk factors, and non-hematological toxicity. *P* <0.05 was considered a statistically significant difference.

## Results

### General clinical data

A total of 112 patients with early-stage cervical cancer (FIGO IB1-IIA2) underwent extensive hysterectomy and pelvic lymph node dissection. The diagnosis was confirmed by postoperative pathology. All of the patients had at least one high-risk factor or two intermediate risk factors. The age, FIGO stage, pathological type, and concurrent chemotherapy of the patients are shown in Table 1.

### Treatment and basic blood levels

Radiation therapy started four weeks after surgery. IMRT treatment was delivered using 6-MV beam in 7 fields. All patients finished their radiation treatment. The average CCRT was four cycles (2-5 cycles). The median values of basal white blood cell count, hemoglobin count, platelet count, and neutrophil count were 5.2×10^9^/L, 123g/L, 261×10^9^/L, and 3.2×10^9^/L, respectively, before the post-operative CCRT.

### Grading and incidence of acute HT

The mean values of the patients' lowest white blood cell, hemoglobin, platelet, and neutrophil counts during the concurrent chemoradiotherapy were 2.25×10^9^/L, 107.3g/L, 131×10^9^/L, and 1.7×10^9^/L, respectively. The incidence of grade 2 or higher leukopenia, hemoglobin reduction, thrombocytopenia, and neutropenia were 49.1%, 2.7%, 1.8%, and 20.5%, respectively (Table 2).

### Dose-Volume analysis of pelvic bone marrow

The results showed that the mean radiation volumes receiving 10Gy, 20Gy, 30Gy, 40Gy, 45Gy, and 50Gy (LS-V10, LSV20, LS-V30, LS-V40, LS-V45, and LS-V50) were generally higher in LS-BM than other parts of the pelvic bone marrow. The dosimetric results are shown in Table 3.

### Correlation analysis results

Leukopenia was associated with LS-V20 (r = -0.310; *P* = 0.006) and length of radiation treatment (days) (r = -0.416; *P* = 0.013), and independent of other factors. Decreased hemoglobin was associated with WP-V30, WP-V40, WP-V45, WP-V50, IL-V20, IL-V40, IL-V45, IL-V50, LP-V30, LP-V40, LP-V45, and LPV50 (*P* <0.05), and unrelated to other factors. Thrombocytopenia (r = -0.304, *P* = 0.007) and neutropenia (r = -0.368, *P* = 0.009) were only related to the length of treatment time (days), and not to other factors (Table 4).

### Results of multiple linear regression analysis

Multiple regression analysis showed that hemoglobin measurement was negatively correlated with WP-V30, IL-V40, and LP-V40 (*P* < 0.05, Table 5) and had no correlation with other factors (age, pathological type, FIGO stage, total radiotherapy dose, radiotherapy duration, total dose of chemotherapy, and frequency of chemotherapy). All other research indicators showed (white blood cell count, platelet count, and neutrophil count) no correlation with the influence factor.

## Discussion

HT is the most common acute adverse event caused by CCRT in early-stage high-risk cervical cancer patients. Isohashi et al. found that the overall incidence of grade 3 and above acute HT was 45% in a study using postoperative radiotherapy combined with cisplatin concurrent chemotherapy for early-stage high-risk cervical cancer 13. In 75 patients who received concurrent cisplatin chemotherapy during radiotherapy, Lewis et al. observed that the incidence of grade 2 and above leukopenia, neutropenia, anemia, and thrombocytopenia were 26%, 40%, 26.5%, and 1.4%, respectively 14. The incidence of grade 3-4 leukopenia, thrombocytopenia, and neutropenia in our study was 5.4%, 0%, and 1.8%, respectively, which was lower than the studies mentioned above.

Studies have shown that the pelvic bone marrow is one of the main hematopoietic sites in the human body. About 50% of the bone marrow is located in the ilium, low pelvis, and lumbosacral vertebrae 15. A large number of studies have shown that HT is closely related to the volume of pelvic bone marrow receiving a radiation dose 16, 17. Kumar et al. investigated the factors that influence the hematological toxicity of cervical cancer radiotherapy combined with cisplatin or carboplatin chemotherapy using a multiple regression model. They discovered a link between grade 4 HT and lower pelvis V5Gy > 95%, lower pelvis V20Gy > 45%, and total pelvic bone V20Gy > 65% (*P* < 0.05) 16. In addition, studies have shown that the dose volume of the LS-BM and lower pelvic bone marrow is more closely related to the occurrence of hematological toxicity than the iliac bone marrow. Albuquerque et al. performed a logistic regression analysis in the study of cisplatin chemotherapy for cervical cancer with 3D-CRT (Three-dimensional Conformal Radiation Therapy) simultaneously and showed that only the low-dose radiation volume, V20Gy of the WP-BM, was significantly associated with grade 2 and above HT. The incidence of grade 2 and above HT increases by 4.5 times when V20Gy is larger than 80% 18. Rose et al. showed that patients with WP-BM low-dose radiation volume, V10Gy larger than 95%, and V20Gy larger than 76% were prone to grade 3 or above HT 19. Klopp et al. analyzed the acute HT of patients in the RTOG0418 clinical trial. They found that when the WP-BM had a high-dose radiation volume, with V40Gy larger than 37% after IMRT, the incidence of grade 2 or above HT increased 20. In addition, studies have shown that acute HT after CCRT for cervical cancer is not only related to the low-dose radiation volume of the pelvis but that different chemotherapy regimens (such as chemotherapy cycles, weekly dose, and total dose) also have varying degrees of impact on the occurrence of HT 21. This study showed that acute HT induced by CCRT after surgery for early-stage high-risk cervical cancer might be related to the length of radiation treatment time (days) and the volume of pelvic bone marrow receiving both low and high doses.

Approaches to reduce the HT of CCRT for cervical cancer patients have become a current research hotspot. With the continuous improvement of radiotherapy, the incidence of HT has decreased significantly. Studies have shown that IMRT significantly reduces the dose and volume of pelvic radiation compared with 3D-CRT 22. IMRT has been proved to considerably reduce the volume of pelvic bone marrow receiving high-dose radiation, such as V30Gy, V40Gy, and V50Gy, as well as the incidence of grade 2 or above acute HT 23.

Standard IMRT technology limits the radiation dose to normal tissues around the tumor, such as the bladder, rectum, and small intestine. Bone Marrow Sparing-Intensity Modulated Radiotherapy (BMS-IMRT) is a type of IMRT-based technique that aims to limit the dose of pelvic bone marrow and proximal femur. The purpose of BMS-IMRT is to protect the pelvic bone marrow, while not compromising the coverage of the target, as well as the sparing of other normal tissues 9, 24. Sun et al. compared the protective effect of IMRT and BMS-IMRT on the pelvic bone marrow in patients after cervical cancer surgery. They found that BMS-IMRT could significantly reduce V10Gy-V50Gy in pelvic bone 9. Huang et al. prospectively studied the effect of BMS-IMRT on HT. The incidence of grade 2 or above HT in the BMS-IMRT group was 50.0%, significantly lower than the IMRT group's 69.5% (P=0.02). Patients were more likely to develop a grade 2 or above HT when V40Gy in pelvic bone larger than 28%. The incidence of grade 2 or above gastrointestinal toxicity did not differ significantly between the two groups 25. In this study, routine IMRT was used, and acute HT still occurred. In the future, large randomized controlled clinical trials are needed to confirm the protective effect of BMS-IMRT on pelvic bone marrow.

There are some shortcomings in this study. Firstly, we included cortical bone when delineating the pelvic bone marrow, which may have weakened the relationship between the pelvic bone marrow and acute HT. Secondly, this study is a retrospective study that only analyzed the influencing factors of acute HT in patients with early-stage high-risk cervical cancer who received external beam radiation therapy. Large, prospective randomized controlled clinical trials are needed to confirm whether BMS-IMRT could reduce acute HT.

In conclusion, our current study suggests that the acute hematological toxicity of IMRT concurrent chemotherapy in patients with early-stage high-risk cervical cancer could be related to the radiation treatment length and the low-dose or high-dose volume received in the pelvic bone marrow.

### Author Contributions

Drs. X. J. Yang and Fu had full access to all data in the study and take responsibility for the integrity of the data and the accuracy of the data analysis.

Concept and design: X.J. Yang, Fu.

Acquisition, analysis, or interpretation of data: All authors.

Drafting of the manuscript: X.J. Yang, Z. Li, and Fu.

Critical revision of the manuscript for important intellectual content: Zhang, Sun, and X.M. Yang.

Statistical analysis: X. J. Yang, and H. Li.

Obtained funding: X. J. Yang.

Administrative, technical, and material support: X.J. Yang, Zhang, Sun, and X.M. Yang.

Supervision: Zhang.

### Ethics approval and consent to participate

All patients signed informed consent. The study was approved by the Ethics Committee of Shanghai Sixth People's Hospital Affiliated to Shanghai Jiao Tong University School of Medicine and followed the Declaration of Helsinki to the letter.

### Data Availability Statement

The data used to support the findings of this study are included within the article.

### Funding

This study was funded by the Shanghai Jiao Tong University Affiliated Sixth People's Hospital (contract grant number: ynqn202118).

### Role of the Funder

The funding organization had no role in the design and conduct of the study; collection, management, analysis, and interpretation of the data; preparation, review, or approval of the manuscript; or decision to submit the manuscript for publication.

## Figures and Tables

**Figure 1:**
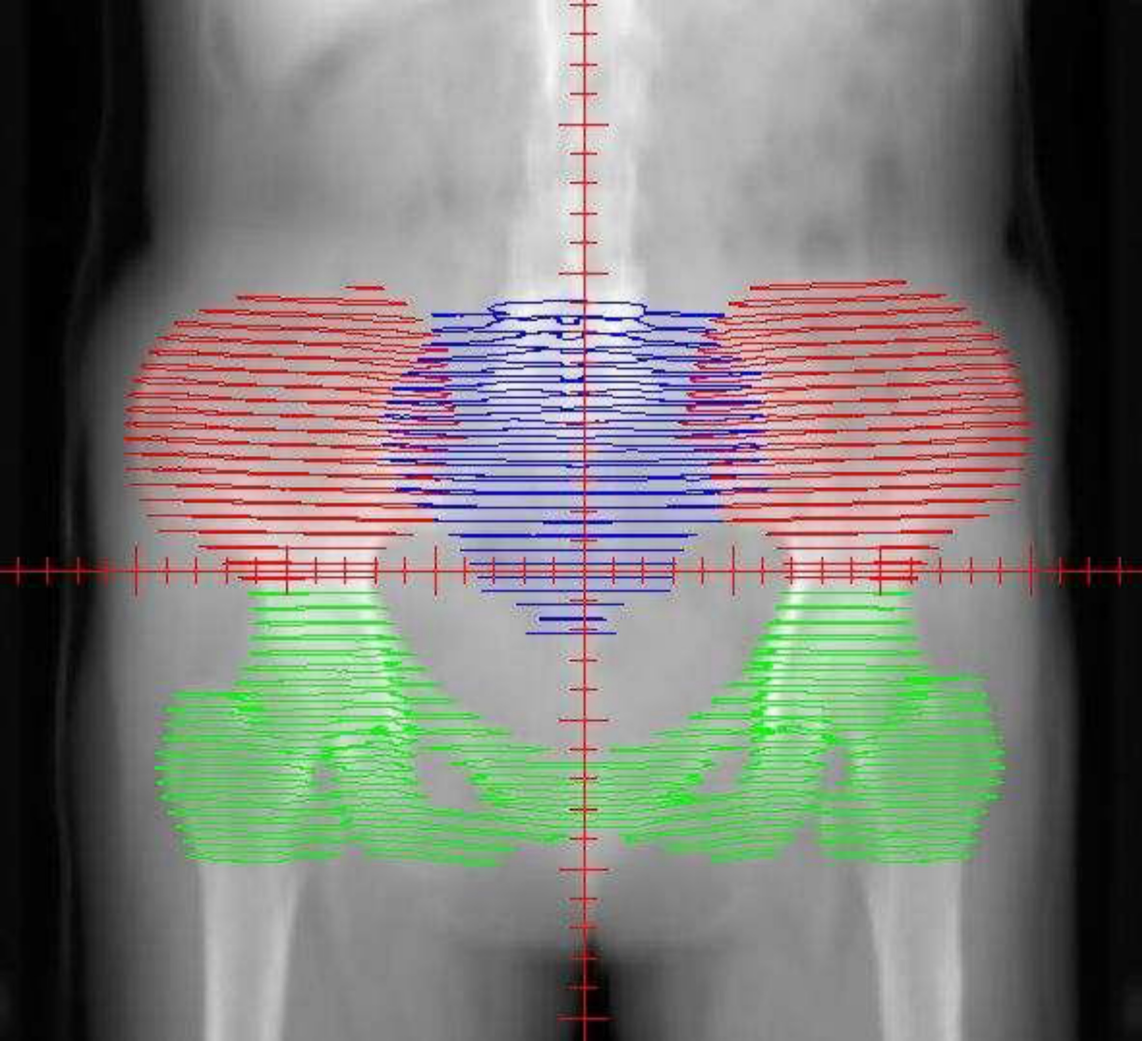
The distribution of the bone marrow in the pelvic. The red area represents the iliac bone marrow (IL-BM). The green area represents the low pelvic bone marrow (LP-BM). The blue area represents the lumbosacral bone marrow (LS-BM).

**Table 1:** Patient characteristics

Subject characteristics	Number of patients	%
Age		
≤50	55	50.89
>50	57	49.11
Histological pattern		
Squamous cell carcinoma	88	78.57
Adenocarcinoma	21	19.75
Adenosquamous carcinoma	2	1.79
Small cell carcinoma	1	0.89
ECOG score		
0	67	59.82
1	39	34.82
2	6	5.36
FIGO stage		
IB 1	25	22.32
IB 2	27	24.11
IB 3	12	10.71
IIA 1	25	22.32
IIA 2	23	20.54
Chemotherapy drugs		
DDP	51	45.54
NDP	61	54.46
Chemotherapy cycles received		
2	8	7.14
3	43	38.39
4	44	39.29
5	17	15.18

**Table 2:** Grading of acute hematological toxicity [number of cases (%)]

Toxicity	Grade 0	Grade 1	Grade 2	Grade 3	Grade 4
Leukopenia	22 (19.6)	35 (31.3)	49 (43.7)	6 (5.4)	0 (0)
Anemia	45 (40.2)	64 (57.1)	3 (2.7)	0 (0)	0 (0)
Thrombocytopenia	95 (84.8)	15 (13.4)	2 (1.8)	0 (0)	0 (0)
Neutropenia	57 (50.9)	32 (28.6)	21 (18.7)	2 (1.8)	0 (0)

**Table 3:** Pelvic bone marrow dosimetry parameters

Parameter	Mean (%)	Standard Deviation
**IL-BM**		
IL-V10	98.40	0.31
IL-V20	85.37	0.77
IL-V30	52.16	0.98
IL-V40	25.83	0.93
IL-V45	15.56	0.70
IL-V50	7.03	0.49
**LP-BM**		
LP-V10	75.78	0.83
LP-V20	61.06	0.91
LP-V30	35.71	0.69
LP-V40	15.65	0.60
LP-V45	9.78	0.45
LP-V50	3.87	0.20
**LS-BM**		
LS-V10	99.90	0.17
LS-V20	97.63	0.39
LS-V30	86.72	1.45
LS-V40	55.76	1.63
LS-V45	39.40	1.04
LS-V50	20.11	1.35
**WP-BM**		
WP-V10	90.87	0.63
WP-V20	81.36	0.67
WP-V30	56.35	0.92
WP-V40	31.03	0.77
WP-V45	21.43	0.83
WP-V50	9.93	0.69

**Table 4:** Relationship between bone marrow exposure dose and hematological toxicity

Parameter	Leukopenia r (*P*)	Anemia r (*P*)	Thrombocytopenia r (*P*)	Neutropenia r (*P*)
IL-V20	0.089 (0.671)	-0.413 (0.018*)	0.230 (0.174)	0.135 (0.641)
IL-V40	0.135 (0.541)	-0.451 (0.007*)	0.211 (0.376)	-0.768 (0.510)
IL-V45	-0.306 (0.412)	-0.396 (0.006*)	0.191 (0.541)	-0.172 (0.510)
IL-V50	-0.216 (0.503)	-0.378 (0.008*)	0.273 (0.509)	-0.177 (0.554)
LP-V30	-0.231 (0.206)	-0.401 (0.006*)	-0.041 (0.761)	-0.115 (0.517)
LP-V40	-0.272 (0.102)	-0.511 (0.002*)	-0.037 (0.917)	-0.186 (0.312)
LP-V45	-0.315 (0.065)	-0.472 (0.003*)	-0.051 (0.961)	-0.201 (0.172)
LP-V50	-0.276 (0.103)	-0.453 (0.004*)	-0.041 (0.698)	-0.213 (0.375)
LS-V20	-0.310 (0.006*)	-0.137 (0.151)	-0.096 (0.515)	-0.376 (0.093)
WP-V30	-0.093 (0.615)	-0.306 (0.021*)	0.155 (0.606)	-0.037 (0.761)
WP-V40	-0.217 (0.450)	-0.453 (0.003*)	0.059 (0.711)	-0.303 (0.402)
WP-V45	-0.311 (0.078)	-0.431 (0.012*)	0.034 (0.780)	-0.091 (0.206)
WP-V50	-0.224 (0.194)	-0.330 (0.031*)	0.057 (0.758)	-0.256 (0.137)
Duration of radiotherapy (days)	-0.416 (0.013*)	0.007 (0.906)	-0.304 (0.007*)	-0.368 (0.009*)

**Table 5:** Multiple linear regression analysis results

	Anemia		
	**β**	**t**	**P**
WP-V30	-14.713	02.455	0.031
IL-V40	-8.754	-2.137	0.045
LP-V40	-7.064	-2.531	0.034
